# Multiple Posttranscriptional Strategies To Regulate the Herpes Simplex Virus 1 vhs Endoribonuclease

**DOI:** 10.1128/JVI.00818-18

**Published:** 2018-08-16

**Authors:** Gillian Elliott, Kathleen Pheasant, Katja Ebert-Keel, Julianna Stylianou, Ashley Franklyn, Juliet Jones

**Affiliations:** a Section of Virology, Department of Microbial Sciences, Faculty of Health & Medical Sciences, University of Surrey, Guildford, United Kingdom; b Section of Virology, Faculty of Medicine Building, Imperial College London, London, United Kingdom; Northwestern University

**Keywords:** HSV-1, PABP, VP16, VP22, endoribonuclease, vhs

## Abstract

The herpes simplex virus 1 (HSV-1) virion host shutoff (vhs) protein is an endoribonuclease that binds to the cellular translation initiation machinery and degrades associated mRNAs, resulting in the shutoff of host protein synthesis. Hence, its unrestrained activity is considered lethal, and it has been proposed that vhs is regulated by two other virus proteins, VP22 and VP16. We have found that during infection, translation of vhs requires VP22 but not the VP22-VP16 complex. Moreover, in the absence of VP22, vhs is not overactive against cellular or viral transcripts. In transfected cells, vhs was also poorly translated, correlating with the aberrant localization of its mRNA. Counterintuitively, vhs mRNA was predominantly nuclear in cells where vhs protein was detected. Likewise, transcripts from cotransfected plasmids were also retained in the same nuclei where vhs mRNA was located, while poly(A) binding protein (PABP) was relocalized to the nucleus in a vhs-dependent manner, implying a general block to mRNA export. Coexpression of VP16 and VP22 rescued the cytoplasmic localization of vhs mRNA but failed to rescue vhs translation. We identified a 230-nucleotide sequence in the 5′ region of vhs that blocked its translation and, when transferred to a heterologous green fluorescent protein transcript, reduced translation without altering mRNA levels or localization. We propose that expression of vhs is tightly regulated by a combination of inherent untranslatability and autoinduced nuclear retention of its mRNA that results in a negative feedback loop, with nuclear retention but not translation of vhs mRNA being the target of rescue by the vhs-VP16-VP22 complex.

**IMPORTANCE** A myriad of gene expression strategies has been discovered through studies carried out on viruses. This report concerns the regulation of the HSV-1 vhs endoribonuclease, a virus factor that is important for counteracting host antiviral responses by degrading their mRNAs but that must be regulated during infection to ensure that it does not act against and inhibit the virus itself. We show that regulation of vhs involves multifaceted posttranscriptional cellular and viral processes, including aberrant mRNA localization and a novel, autoregulated negative feedback loop to target its own and coexpressed mRNAs for nuclear retention, an activity that is relieved by coexpression of two other virus proteins, VP22 and VP16. These studies reveal the interplay of strategies by which multiple virus-encoded factors coordinate gene expression at the time that they are needed. These findings are broadly relevant to both virus and cellular gene expression.

## Abstract (precis)

A myriad of gene expression strategies has been discovered through studies carried out on viruses. This report concerns the regulation of the HSV-1 vhs endoribonuclease, a virus factor that is important for counteracting host antiviral responses by degrading their mRNAs but that must be regulated during infection to ensure that it does not act against and inhibit the virus itself. We show that regulation of vhs involves multifaceted posttranscriptional cellular and viral processes, including aberrant mRNA localization and a novel, autoregulated negative feedback loop to target its own and coexpressed mRNAs for nuclear retention, an activity that is relieved by coexpression of two other virus proteins, VP22 and VP16. These studies reveal the interplay of strategies by which multiple virus-encoded factors coordinate gene expression at the time that they are needed. These findings are broadly relevant to both virus and cellular gene expression.

## INTRODUCTION

Many viruses encode endoribonucleases which promote the degradation of host mRNAs to block host gene expression, resulting in translational shutoff during infection (1). The endoribonuclease encoded by the UL41 gene of herpes simplex virus 1 (HSV-1), termed vhs (virion host shutoff) (2–5), cleaves RNA in a nonselective fashion *in vitro* (4, 6, 7) but during infection is believed to be targeted specifically to translating mRNA by binding to the cellular translation machinery through the eukaryotic initiation factor 4F cap binding complex and cleaving the associated transcripts (8–11). Subsequent degradation of cleaved host cell mRNA by the cellular 5′-to-3′ exonuclease Xrn1 then occurs (12) with the concomitant abrogation of host protein translation, thereby freeing up host ribosomes to be recruited to virus mRNAs and translate virus proteins. As its name suggests, vhs is packaged into the virion (3, 13) and is proposed to degrade target cellular mRNAs at the very early stages of infection (14, 15). Newly synthesized vhs is expressed with late kinetics, with a significant global reduction in host cell mRNAs being readily detectable at this time (16).

One paradox that surrounds the activity of vhs is its apparent lack of selectivity for cellular mRNAs over viral mRNAs (17). Indeed, vhs has been shown to play a role in the transition from immediate early (IE) to early (E) gene expression by actively degrading IE mRNAs (18, 19), and in cells infected with a virus lacking vhs (Δvhs virus), IE mRNAs are present at higher levels than in wild-type (Wt)-infected cells (20). Hence, because of its ultimately lethal activity, it is considered that high levels of vhs protein, such as those detected at later times of infection, would be generally detrimental to virus infection: multiple virus mRNAs themselves would be degraded by vhs, leading to the eventual total shutoff of virus protein synthesis. Related to this, the phenotypes of viruses deleted for either of the two tegument proteins, VP16 or VP22, have uncovered a potential role for each of them in the regulation of vhs activity, with a translational shutoff phenotype being a consequence of deletion of either of these genes (21–23). As both vhs and VP22 can bind to VP16 (24–26), it has been proposed that these three proteins form a trimeric complex that is required to neutralize the RNase activity of vhs and that in the absence of either VP22 or VP16, vhs ultimately degrades virus mRNA in an unrestrained fashion, leading to the complete shutoff of virus protein synthesis (21, 22, 27). In addition to this role in dampening vhs activity, VP16 and VP22 have also been implicated in enhancing expression of vhs for assembly into virions (28). However, although one model proposes that vhs must be mutated for a VP22 knockout virus to even be viable (27), we and others have reported VP22 deletion mutants that maintain a parental vhs sequence and yet replicate in culture (29, 30).

In the studies presented here, we set out to clarify how the expression of this potentially lethal viral endoribonuclease is regulated to maintain the sensitive balance between cellular mRNA destruction and viral mRNA translation. We show that the vhs mRNA is inherently untranslatable because of the presence of a specific repressive sequence(s) in its open reading frame (ORF) which inhibits vhs translation when it is expressed in isolation but is relieved during virus infection through a process that requires VP22. Moreover, mRNA fluorescent in situ hybridization (FISH) studies revealed that when translation of vhs occurred in transfected cells, it exerted negative feedback on its own and other transcripts to cause retention in the nucleus, an activity that was also marked by the nuclear retention of poly(A) binding protein (PABP). Such nuclear retention limits the extent of vhs endoribonuclease activity while also blocking translation of the retained heterologous mRNAs. Importantly, it is nuclear retention and not translation of vhs mRNA that is relieved by coexpression of VP16 and VP22, an effect that extends to coexpressed mRNAs.

## RESULTS

### Efficient translation of vhs in HSV-1-infected cells requires VP22 but not the VP22-VP16 complex.

**FIG 1:**
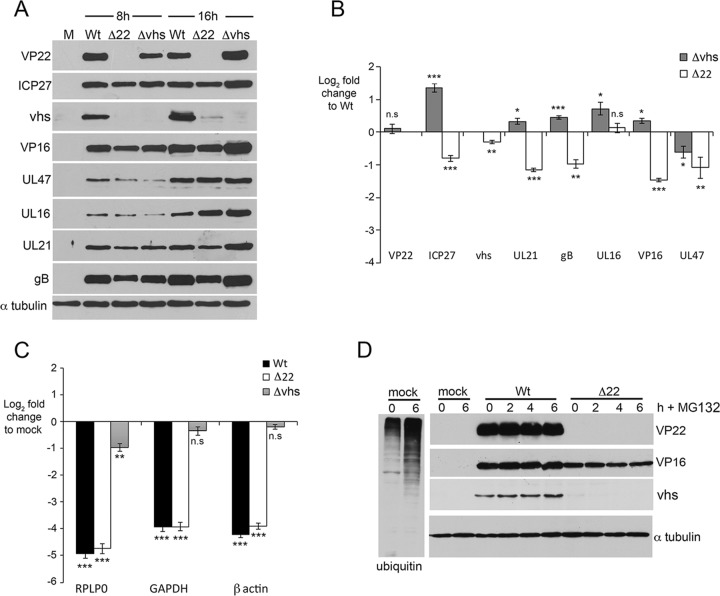
Efficient translation of HSV-1 vhs requires VP22 in HSV-1-infected cells. (A) HeLa cells were infected with Wt (s17), Δ22, or Δvhs viruses at a multiplicity of 2 or left uninfected (lane M) and harvested at 8 and 16 h after infection for Western blotting with antibodies, as indicated. (B) As for panel A, but total RNA was purified from cells harvested at 16 h after infection and processed for qRT-PCR with the indicated primer sets. Transcript levels are expressed in relation to those found in Wt-infected cells. The *y* axis represents the log_2_ fold change in expression from that in Wt-infected cells. The mean ± standard error of the data from one representative experiment is given (*n* = 3). Statistical analysis was carried out using an unpaired, two-way Student's *t* test. (C) The RNA samples from the assay whose results are presented in panel B were processed for qRT-PCR with the indicated primer sets for cell housekeeping genes. Transcript levels are expressed in relation to those found in uninfected cells, and the *y* axis represents the log_2_ fold change in expression compared to that in uninfected cells. The mean ± standard error of the data from one representative experiment is given (*n* = 3). Statistical analysis was carried out using an unpaired, two-way Student's *t* test. (D) HeLa cells infected with Wt or Δ22 viruses at a multiplicity of 2 or left uninfected (lanes mock) were incubated for 14 h before adding fresh medium with or without 10 μM MG132. Samples were then harvested every 2 h for a further 6 h and analyzed by Western blotting for the indicated proteins. MG132 activity was confirmed by blotting uninfected cell extracts for polyubiquitin. n.s., not significant. *, *P* < 0.05; **, *P* < 0.01; ***, *P* < 0.001.

We have previously shown that vhs is present at very low, often undetectable levels in cells infected with a virus lacking the VP22 gene (Δ22 virus) (30), a feature that might be expected to result in reduced vhs activity. However, somewhat counterintuitively, it has also been suggested that in the absence of VP22, vhs endoribonuclease activity is overactive, resulting in the enhanced degradation of viral mRNAs and the subsequent shutoff of virus translation (22, 27). To clarify the status and activity of vhs during Δ22 infection, we infected HeLa cells with Wt, Δ22, or Δvhs viruses, harvested total cell extracts at 8 and 16 h after infection, and analyzed these by Western blotting for a number of key virus proteins (Fig. 1A). As shown previously, vhs was hardly detectable in the Δ22-infected cell lysates at either time point (Fig. 1A), but, in contrast, the other proteins tested—ICP27, VP16, UL47, UL16, UL21, and glycoprotein B (gB)—were all expressed at detectable levels close to those in cells infected with the Wt (Fig. 1A). Likewise, virus protein expression in cells infected with the Δvhs virus was similar to that in Wt-infected cells, and for some proteins, such as ICP27 and VP16, expression appeared to be enhanced compared to that in Wt-infected cells (Fig. 1A). We performed quantitative reverse transcription-PCR (qRT-PCR) on infected cells harvested 16 h after infection to determine the relative level of the corresponding mRNAs, using 18S RNA to normalize the levels, thereby avoiding any potential issues with the vhs-specific degradation of housekeeping gene mRNAs. This showed that most of the virus transcripts were reduced by no more than 2-fold in Δ22-infected cells compared to Wt-infected cells (Fig. 1B). Of note, despite the failure to detect the vhs protein in Δ22-infected cells, the mRNA level for vhs was only slightly reduced compared to the mRNA level in Wt-infected cells, indicating that the vastly reduced protein expression of vhs in the absence of VP22 was not a consequence of reduced amounts of mRNA. In contrast, in Δvhs virus-infected cells, most of the virus transcripts tested were present at levels slightly higher than those in Wt-infected cells, with ICP27 being increased about 2.5-fold compared to that in Wt-infected cells (Fig. 1B). This correlates with the findings of previous studies (19, 20).

To determine the relative activity of vhs on cellular transcripts in Δ22-infected cells, we also performed qRT-PCR on the same samples to measure the relative level of three housekeeping genes, RPLP0, GAPDH (glyceraldehyde-3-phosphate dehydrogenase), and β-actin. As anticipated, in Δvhs virus-infected cells, these cellular transcripts remained at a level similar to those found in uninfected cells, reflecting the lack of vhs activity in this infection (Fig. 1C). In contrast, all three transcripts were reduced by between 15- and 30-fold in both Wt- and Δ22-infected cells, presumably as a consequence of vhs degradation of these host mRNAs. There was no significant difference between mRNA degradation in Wt- and Δ22-infected cells, suggesting that, in contrast to the currently accepted model (27), there is no evidence of uncontrolled vhs activity against viral or cellular transcripts in the absence of VP22 in infected HeLa cells.

We next considered if the low protein level of vhs in Δ22-infected cells was a consequence of protein instability. HeLa cells were infected with Wt or Δ22 viruses and after 14 h were incubated for a further 6 h in the absence or presence of the proteasome inhibitor MG132 to enable the accumulation of proteins that are ordinarily degraded through the proteosome. Blotting for polyubiquitin indicated that the inhibitor was functional, as there was an increased level of polyubiquitinated species in the cell lysate from MG132-treated cells (Fig. 1D, ubiquitin blot). Nonetheless, there was no recovery of vhs protein during the course of MG132 treatment, indicating that low vhs expression was unlikely to be a consequence of protein degradation (Fig. 1D).

**FIG 2:**
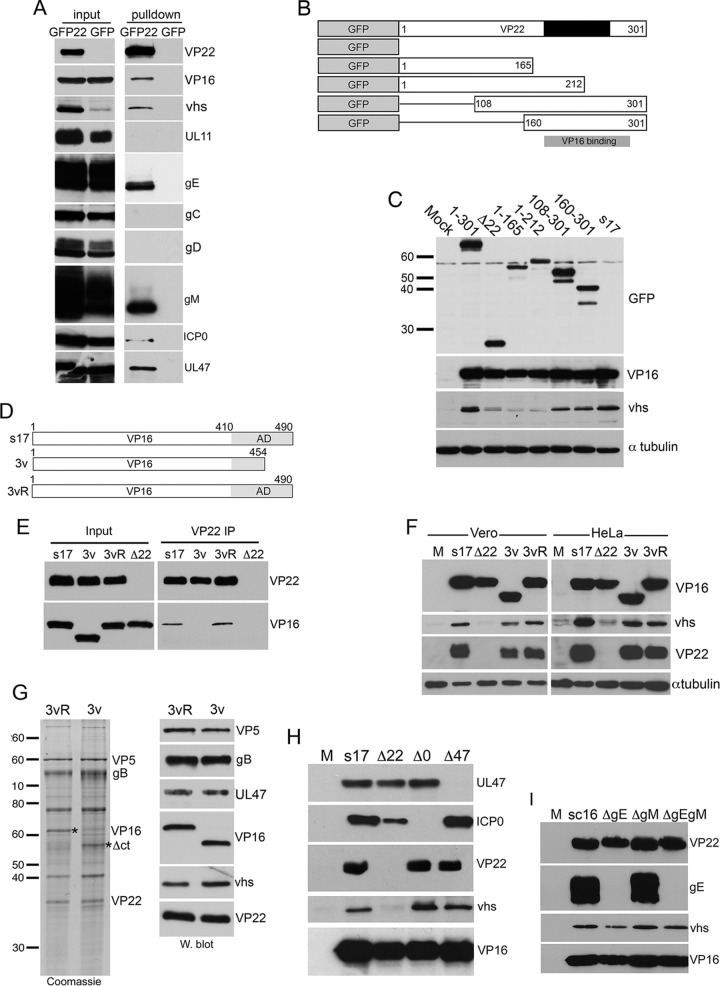
Requirement for VP22 binding partners in vhs expression. (A) VP22 binding proteins were copurified by conducting GFP-TRAP pulldown of Vero cells infected with HSV-1 expressing GFP-VP22 (GFP22) and harvested 16 h after infection at a multiplicity of 2. HSV-1 expressing GFP in place of VP22 was used as a control. The resulting VP22 interactome was analyzed by Western blotting with the indicated antibodies. (B and C) Vero cells were infected with the denoted recombinant viruses based on HSV-1 strain 17 expressing GFP-tagged variants of VP22. Cells were harvested after 16 h, and total extracts were analyzed by Western blotting for GFP, VP16, or vhs. Blotting for α-tubulin indicates equivalent loading of samples. The Wt was HSV-1 strain 17 (s17). (D) Line drawing showing truncated VP16 expressed in the 3v recombinant virus. AD, activation domain of VP16. (E) Vero cells were infected with the Wt (s17), the 3v virus, the revertant of 3v (3vR), or the Δ22 virus at a multiplicity of 2, harvested 16 h later, and subjected to immunoprecipitation (IP) with the VP22 antibody. The resulting complexes were analyzed by Western blotting for VP16 and VP22. (F) Vero and HeLa cells were infected with the Wt (s17), the 3v virus, the revertant of 3v (3vR), or the Δ22 virus at a multiplicity of 2 and harvested 16 h later, and total lysates were analyzed by Western blotting with the indicated antibodies. (G) Extracellular virions were purified from cells infected with the 3v or 3vR virus, and equivalent amounts were analyzed by SDS-PAGE followed by Coomassie blue staining (left) or Western blotting with the indicated antibodies (right). (H and I) HeLa cells were infected with the Wt, Δ22, Δ47, and ΔICP0 viruses (H) or Wt, ΔgE, ΔgM, or ΔgEgM viruses (I) at a multiplicity of 2, harvested 16 h after infection, and analyzed by Western blotting with the indicated antibodies. The numbers to the left of the gels in panels C and G are molecular masses (in kilodaltons). Lanes M in panels F, H, and I, mock-transfected cells.

Both VP22 and vhs are known to bind to VP16 (24, 25), and it has been proposed before that this trimeric complex of vhs, VP22, and VP16, mediated by VP16, is required for the accumulation of the vhs protein in the cell (28). To confirm that both these partners copurify with VP22, green fluorescent protein (GFP)-TRAP pulldown was carried out from Vero cells infected with virus expressing GFP fused to full-length VP22 or virus expressing GFP in place of VP22 and Western blotting for a range of virus proteins. This confirmed that both VP16 and vhs were present in the VP22 interactome, together with its known glycoprotein partners, gE and gM, and other proposed partners, ICP0 and UL47, whereas the absence of UL11 and the glycoproteins gD and gC confirmed the specificity of the pulldown (Fig. 2A). Of note, analysis of vhs expression in a range of viruses expressing VP22 truncations tagged with GFP (Fig. 2B) indicated that vhs expression requires the region of VP22 that contains the VP16 binding domain (Fig. 2C).

To determine if the VP22-VP16 complex is required for vhs expression, we utilized a virus lacking the C-terminal 36 residues of the VP16 activation domain (virus 3v; Fig. 2D), the region of VP16 known to interact with VP22 (25, 26). Vero and HeLa cells were infected with 3v and its revertant, 3vR (Fig. 2D), alongside Δ22 and its Wt (strain 17 [s17]) and harvested for immunoprecipitation 16 h later. The lack of interaction between VP22 and VP16 in the 3v infection was confirmed by immunoprecipitation of VP22 from infected cells and Western blotting for VP16, revealing efficient copurification of full-length VP16 with VP22 but not truncated VP16 (Fig. 2E). Nonetheless, analysis of total cell lysates by Western blotting showed that despite the absence of a VP16-VP22 complex in 3v-infected cells, vhs was expressed to the Wt level in this virus infection compared to its level of expression in Δ22 infection (Fig. 2F). These results indicate that in HeLa and Vero cells at least, VP22 but not the VP22-VP16 complex is required for the translation of vhs. Furthermore, purification and analysis of 3v virus particles showed that these virions assembled truncated VP16, vhs, and VP22 to the same level as virions from its revertant virus, 3vR, indicating that a tripartite VP16-VP22-vhs complex is not required to recruit any of these proteins into the virus particle (Fig. 2G). Taken together, these results indicate that the VP22-VP16 interaction is not required for translation of vhs and that the C-terminal half of VP22 plays an altogether different and VP16-independent role in regulating the accumulation of vhs protein. Finally, analysis of HeLa cells infected with viruses defective in other components of the VP22 interactome (gE, gM, ICP0, and UL47) showed that vhs was efficiently expressed in all of these infections, and hence, none of these VP22 binding partners is required for vhs translation (Fig. 2H and I).

### The HSV-1 vhs protein is expressed at low levels in the absence of virus infection.

**FIG 3:**
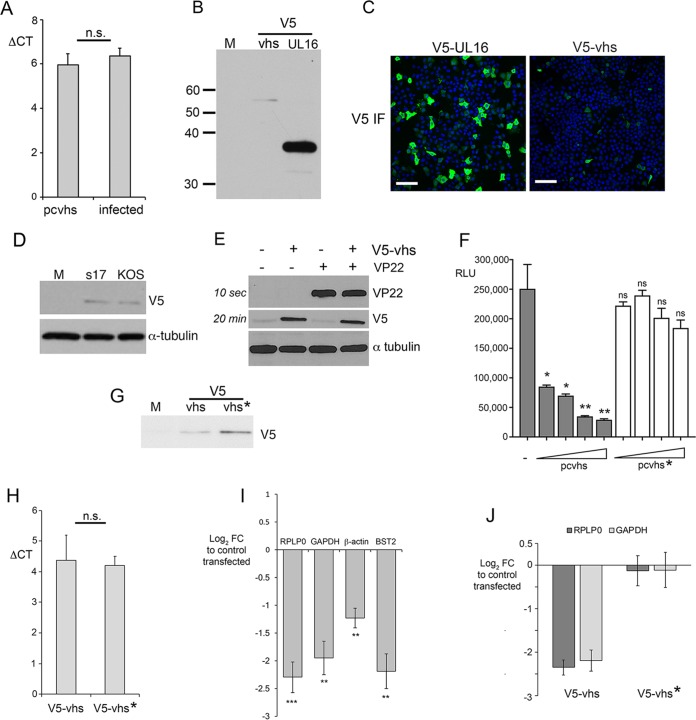
Expression of HSV-1 vhs is restricted at a posttranscriptional stage in the absence of virus infection. (A) Total RNA was purified from HeLa cells transfected with a plasmid expressing untagged vhs (pcvhs) and harvested 20 h later or infected with Wt virus and harvested 16 h after infection. qRT-PCR was carried out for vhs, with transcript levels being represented as the Δ*C_T_* from the reference 18S RNA level. The mean ± standard error of the data from one representative experiment is given (*n* = 3). Statistical analysis was carried out using an unpaired, two-way Student's *t* test. (B and C) HeLa cells transfected with a plasmid expressing V5-vhs or V5-UL16 were analyzed by Western blotting (B) or immunofluorescence (IF) (C) with the V5 antibody. (D) HeLa cells transfected with a plasmid expressing V5-vhs from strain 17 or strain Kos were analyzed by Western blotting with the V5 antibody. (E) HeLa cells transfected with a plasmid expressing vhs in the absence or presence of a plasmid expressing VP22 were analyzed by SDS-PAGE and Western blotting with the indicated antibodies. Note in this instance that the V5 blot was exposed to film for 20 min. (F) HeLa cells were transfected with a plasmid expressing Gaussia luciferase (GLuc), together with increasing amounts of the pcvhs plasmid or the pcvhs* plasmid. After 20 h the medium was changed and then harvested 3 h later, and the level of GLuc was measured. The mean ± standard error of the data from one representative experiment is given (*n* = 3). Statistical analysis was carried out using an unpaired, two-way Student's *t* test. RLU, relative light units. (G) HeLa cells transfected with a plasmid expressing V5-vhs or V5-vhs* with an R257C variation were analyzed by Western blotting with the V5 antibody. (H) Total RNA was purified from HeLa cells transfected with a plasmid expressing V5-vhs or V5-vhs* and harvested 20 h later, and qRT-PCR was carried out for vhs, with transcript levels being represented as the Δ*C_T_* from the reference 18S RNA level. The mean ± standard error of the data from one representative experiment is given (*n* = 3). Statistical analysis was carried out using an unpaired, two-way Student's *t* test. (H) (I) RNA samples were purified from HeLa cells transfected with a plasmid expressing glycoprotein D (pcgD) or vhs (pUL41) and were analyzed by qRT-PCR for the cell housekeeping genes, as indicated. Values obtained are represented as the log_2_ fold change (FC) from the value for cells transfected with the gD-expressing plasmid. The mean ± standard error of the data from one representative experiment is given (*n* = 3). Statistical analysis was carried out using an unpaired, two-way Student's *t* test. (J) RNA samples were purified from HeLa cells transfected with a plasmid expressing V5-vhs or V5-vhs* and analyzed by qRT-PCR for the cell housekeeping genes, as indicated. Values obtained are represented as the log_2_ fold change from the value for cells transfected with the vector alone. The mean ± standard error of the data from one representative experiment is given (*n* = 3). Statistical analysis was carried out using an unpaired, two-way Student's *t* test. Lanes M in panels B, D, and G, mock-transfected cells. *, *P* < 0.05; **, *P* < 0.01; ***, *P* < 0.001.

It has been reported previously that the vhs protein fails to accumulate in cells when expressed in isolation from virus infection by transient transfection (5, 28). After transfection of a plasmid that expresses the vhs open reading frame from HSV-1 strain 17 under the control of the human cytomegalovirus immediate early promoter (pcvhs) into HeLa cells, COS-1 cells, or Vero cells, we were unable to detect vhs expression by Western blotting with the vhs polyclonal antibody available to us (data not shown). Nonetheless, qRT-PCR revealed that the level of vhs mRNA was similar in transfected and infected cells (Fig. 3A). To increase the sensitivity of vhs detection in transfected cells, Western blotting and immunofluorescence were carried out on HeLa cells transfected with a plasmid expressing either V5-tagged vhs or a V5-tagged control protein, in this case, UL16. This indicated that a fusion protein of the correct size for V5-vhs was barely detectable by Western blotting in comparison to the control V5-UL16 protein (Fig. 3B), while V5 immunofluorescence indicated that V5-vhs was expressed in only a small subset of cells compared to V5-UL16 (Fig. 3C), despite both fusion proteins being expressed from the same plasmid backbone. This poor expression was not specific to strain 17 vhs, as vhs from strain Kos was similarly difficult to detect when expressed by transient transfection (Fig. 3D). Nonetheless, despite the requirement for VP22 during infection, coexpression of VP22 with vhs during transient transfection failed to rescue the translation levels of vhs (Fig. 3E).

In the process of isolating a revertant for our Δ22 virus, we identified a variant of vhs containing a R257C substitution (30), a change which, according to other studies, may have come about to inactivate vhs in the absence of VP22 (22, 27). To test the ability of Wt and R257C vhs (referred to as vhs*) to reduce expression of a reporter protein by transfection, increasing amounts of plasmids expressing either form of vhs were cotransfected with a plasmid expressing the reporter Gaussia luciferase (GLuc) (Fig. 3F). In this case, Wt vhs reduced expression of GLuc by up to 10-fold (Fig. 3F, pcvhs), while vhs with the R257C change had no effect on coexpressed GLuc (Fig. 3F, pcvhs*), suggesting that it is defective in vhs activity. Nonetheless, when tested by Western blotting, we found that this variant, vhs*, was translated at a similarly low level as Wt vhs (Fig. 3G), while qRT-PCR indicated similar levels of vhs mRNA to the Wt (Fig. 3H), suggesting that, although it is defective, this variant of vhs was expressed at equivalent levels as Wt vhs in transient transfection.

To determine directly the activity of transfected vhs against cellular mRNAs, the relative level of RPLP0, GAPDH, and β-actin mRNAs in cells transfected with a plasmid expressing vhs was measured using qRT-PCR and compared to that in cells expressing an alternative protein from the same plasmid backbone, in this case, gD (Fig. 3I). At the same time, we also measured the relative level of tetherin (BST-2) mRNA, as this is one of the previously published targets of vhs activity (31). vhs expressed by transfection was capable of reducing each of these mRNAs by up to 4-fold in comparison to the its level in cells expressing gD. In contrast, vhs* had little effect on the level of RPLP0 or GAPDH mRNAs (Fig. 3J; compare vhs with vhs*), confirming that this variant is defective for vhs effects on transcript levels.

### Expression of vhs causes nuclear retention of its own and coexpressed transcripts.

**FIG 4:**
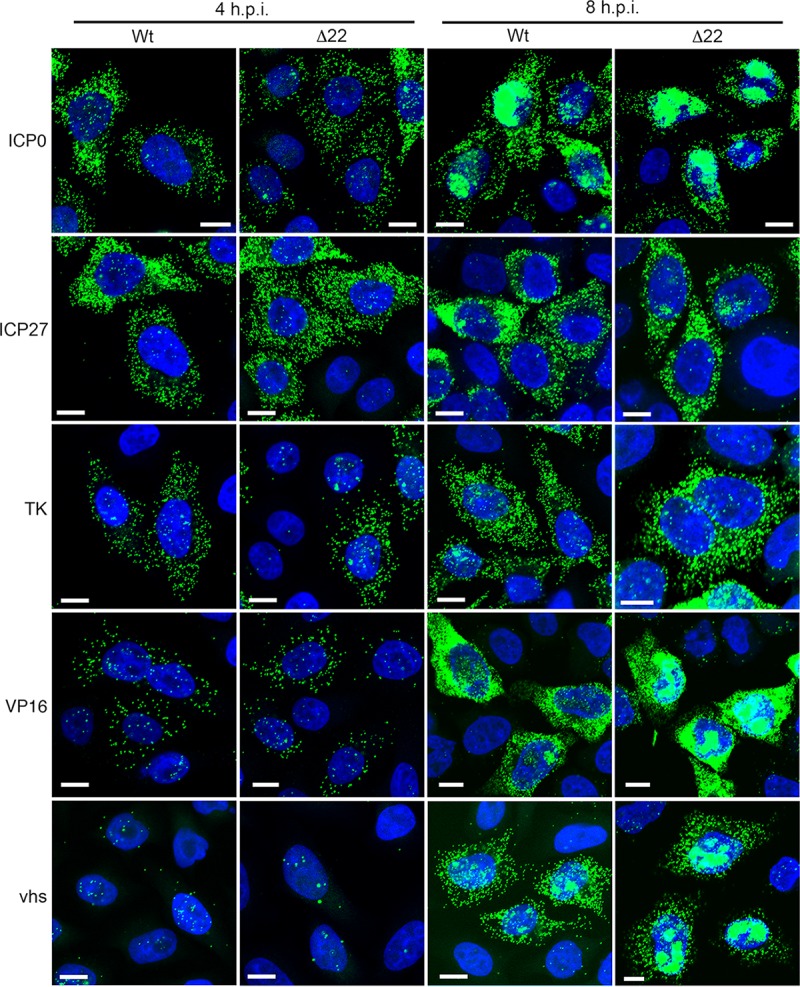
Subcellular localization of HSV-1 transcripts in infected HeLa cells. HeLa cells were infected with Wt (s17) or HSV-1 Δ22 at a multiplicity of 2, fixed at 4 or 8 h after infection, and processed for RNA FISH with probes for ICP0, ICP27, TK, VP16, or vhs mRNA (green). p.i., postinfection. Bars = 10 μm.

A potential explanation for the lack of translation of vhs is that its mRNA is mislocalized and is inaccessible to the translation machinery. To examine this, we carried out fluorescent *in situ* hybridization (FISH) of HeLa cells infected with either Wt or Δ22 viruses and fixed at 4 h or 8 h after infection and compared the localization of five virus transcripts: ICP0 and ICP27 as representative immediate early mRNAs; TK as a representative early mRNA; and VP16 and vhs itself, which are expressed with late kinetics. The specificity of this assay was first tested by pretreating Wt-infected cell samples that had been fixed at 8 h with either DNase or RNase prior to incubation with the ICP27-specific fluorescent probe, revealing a loss of signal after RNase treatment but not after DNase treatment (data not shown). At 4 h, the FISH pattern of all five mRNAs was similar in number and localization between Wt- and Δ22-infected cells, with ICP0 and ICP27 being present in numerous cytoplasmic puncta, indicative of their IE kinetics. Importantly, the number of fluorescent puncta was representative of the known kinetics of each gene examined (Fig. 4, 4 h). At 8 h, the ICP27 and TK transcripts were still predominantly cytoplasmic, but a large number of ICP0 transcripts were now localized to the nucleus, a result taken to be indicative of the ICP27-induced preferential export of nonspliced mRNAs (32) (Fig. 4, 8 h). It was noteworthy that the numbers of ICP0, ICP27, and TK transcripts had not increased by much between 4 h and 8 h, presumably reflecting the relative kinetics of these mRNAs. Nonetheless, there was no obvious difference in these IE transcripts between Wt- and Δ22-infected cells. VP16 and vhs transcripts were more numerous by 8 h, and although they were predominantly cytoplasmic in Wt infection, they had enhanced distinct nuclear populations in the Δ22-infected cells (Fig. 4, 8 h). This suggested that in the absence of VP22, at least two late transcripts, including vhs itself, were prone to nuclear retention. However, given that we had shown above that vhs but not VP16 was poorly translated in the absence of VP22 at 8 h in infected HeLa cells (Fig. 1A), this nuclear retention was not sufficient to explain the lack of translation in Δ22-infected cells.

**FIG 5:**
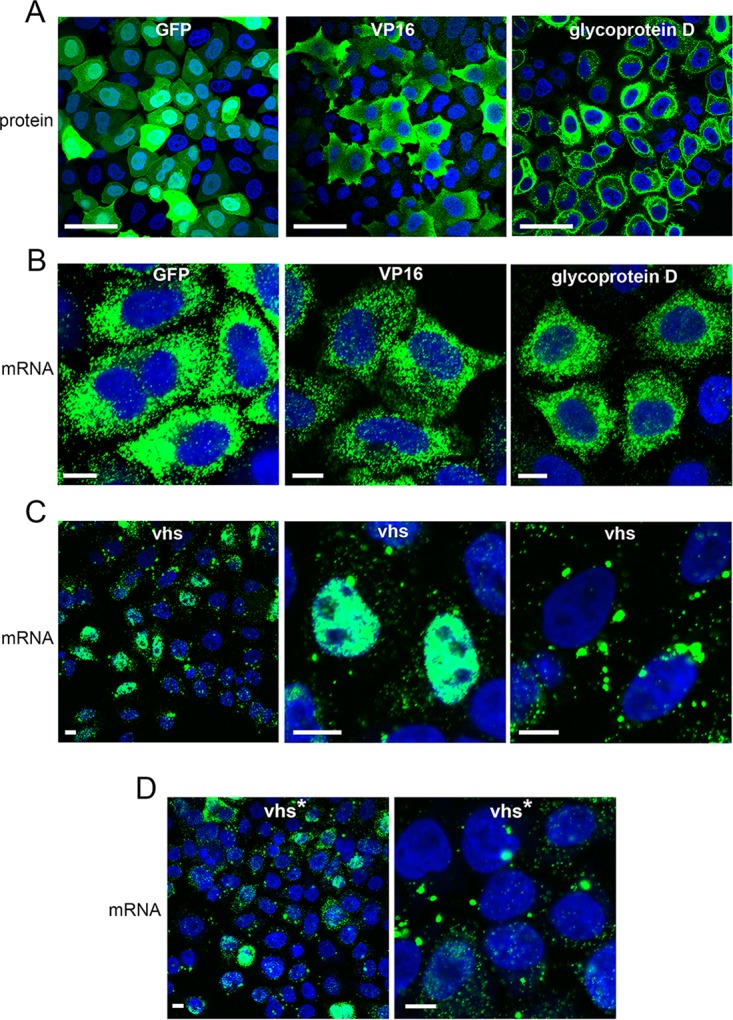
Aberrant localization of vhs mRNA expressed by transient transfection. (A) HeLa cells transfected with plasmids expressing GFP, VP16, or gD were analyzed for protein expression by fluorescence for GFP or immunofluorescence for VP16 and gD (green). (B, C, and D) HeLa cells transfected with plasmids expressing GFP, VP16, or gD (B), vhs (C), or vhs* (D) were treated with DNase and analyzed by RNA FISH with probes specific for mRNAs, as denoted (green). Bars = 50 μm (A) and 10 μm (B, C, and D).

**FIG 6:**
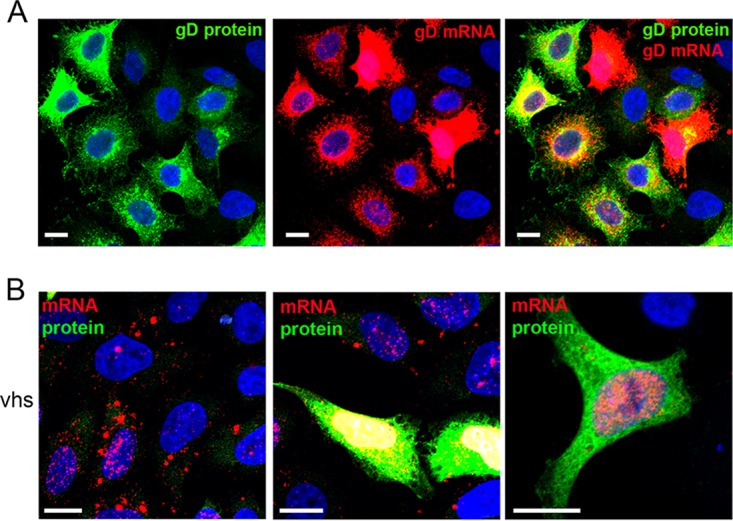
HeLa cells transfected with a plasmid expressing gD (A) or V5-vhs (B) were treated with DNase and incubated for RNA FISH with probes specific for gD or vhs (red), followed by immunofluorescence with a monoclonal antibody to gD or the V5 tag (green). Bars = 10 μm.

FISH was also carried out on transfected HeLa cells to compare vhs mRNA localization when expressed in isolation to that of the highly translated GFP, VP16, or gD mRNA, the last two of which were expressed from the same plasmid backbone as vhs. As expected, GFP, VP16, and gD were efficiently translated in transfected cells, as measured by GFP fluorescence or immunofluorescence for VP16 and gD (Fig. 5A), while their mRNAs were located in numerous cytoplasmic puncta (Fig. 5B). Strikingly, the localization of the vhs mRNA was very different from that of these cytoplasmic puncta of efficiently translated GFP, VP16, or gD transcripts, being either located in cytoplasmic granules or predominantly nuclear (Fig. 5C; compare vhs with GFP, VP16, and gD). Moreover, the mRNA for the vhs* variant was localized to the cytoplasmic granule pattern but not the nuclear pattern (Fig. 5D). To correlate mRNA localization with translation, we combined mRNA FISH with immunofluorescence for either gD or V5-vhs. While gD translation generally correlated with cytoplasmic mRNA (Fig. 6A), in contrast, the V5-vhs protein was not present in the cells where its mRNA was localized to cytoplasmic granules (Fig. 6B) but, counterintuitively, was detectable in the population of cells where vhs mRNA was almost entirely nuclear (Fig. 6B).

**FIG 7:**
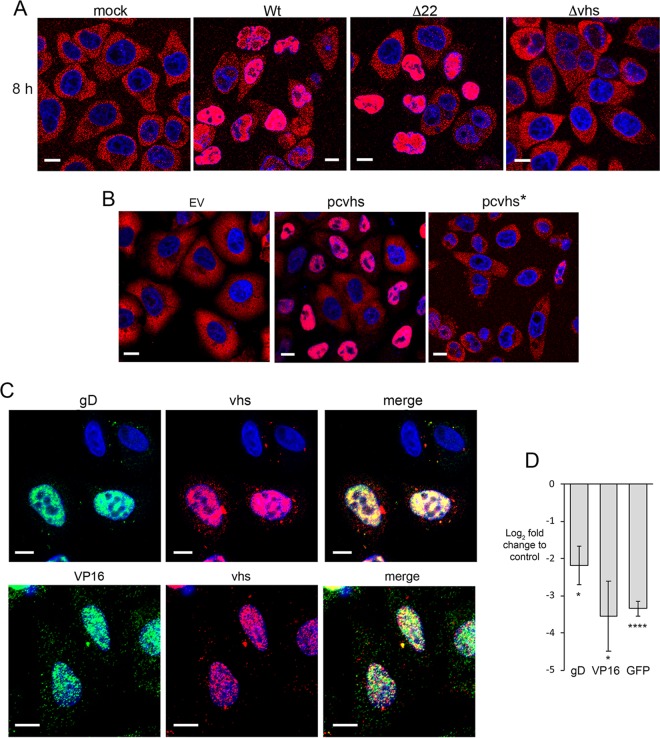
Expression of vhs by transient transfection results in inhibition of mRNA export from the nucleus. (A) Mock-infected or Wt-, Δ22-, or Δvhs-infected HeLa cells were fixed at 8 h and processed for immunofluorescence with a PABP antibody (red). (B) HeLa cells were transfected with an empty vector (EV), a plasmid expressing vhs, or a plasmid expressing vhs*, fixed 20 h later, and processed for immunofluorescence with an antibody to PABP (red). (C) HeLa cells were transfected with a plasmid expressing vhs, together with a plasmid expressing VP16 or gD, fixed 20 h later, and processed for dual mRNA FISH with the appropriate probes. Bars = 10 μm. (D) HeLa cells were transfected with plasmids expressing gD, VP16, or GFP in the absence or presence of a plasmid expressing vhs. After 20 h, total RNA was purified and processed for qRT-PCR, and the values obtained are represented as the log_2_ fold change from the value for cells transfected with an empty expression vector. The mean ± standard error of the data from one representative experiment is given (*n* = 3). Statistical analysis was carried out using an unpaired, two-way Student's *t* test. *, *P* < 0.05; ****, *P* < 0.0001.

A potential explanation for the nuclear phenotype of vhs mRNA is that, once it is translated, vhs protein exerts a negative feedback on its own mRNA and blocks its export from the nucleus. It has been shown before that poly(A) binding protein (PABP)—a protein that shuttles through the nucleus to bind and export polyadenylated mRNAs (33)—relocalizes from its normal steady-state localization in the cytoplasm to a predominantly nuclear location in both HSV-1-infected cells (34, 35) and Kaposi's sarcoma herpesvirus (KSHV)-infected cells (36, 37). In KSHV, this activity has been attributed to its viral alkaline exonuclease (Sox) protein (36–38), although the ORF10 protein has also been implicated (39). In the case of HSV-1, there is evidence that expression of ICP27 alone can induce the nuclear accumulation of PABP (35). However, when we tested PABP localization during infection, we found that its relocalization to the nucleus was similar in Wt- and Δ22-infected cells but was dependent on the expression of vhs (Fig. 7A). Furthermore, and in agreement with work from Kumar and Glaunsinger (38), staining of HeLa cells expressing vhs by transfection revealed that, despite low expression levels, vhs was sufficient to cause the relocalization of PABP to the nucleus of a proportion of cells (Fig. 7B).

Nuclear localization of PABP has been shown to correlate with a general block to mRNA export (40), so we reasoned that other recently transcribed mRNAs expressed after the translation of vhs may also be retained in the nucleus. To test this, we cotransfected the vhs-expressing plasmid into HeLa cells with plasmids expressing gD or VP16 and carried out dual FISH for the coexpressed transcripts. In cells where the vhs mRNA was nuclear—and, hence, by inference from our results presented above were likely to be expressing vhs protein—both gD and VP16 transcripts were retained in the nucleus (Fig. 7C), with a corresponding drop in relative mRNA levels (Fig. 7D). Likewise, coexpression of vhs with GFP resulted in a similar drop in the level of GFP mRNA, confirming that this activity was not specific to viral genes (Fig. 7D). These data suggest that when vhs is translated in transfected cells, its activity on cytoplasmic transcripts, including its own, exerts a negative feedback on the export of newly transcribed mRNAs from the nucleus.

### Coexpression of VP22 and VP16 relieves vhs-induced nuclear retention of its own and heterologous mRNAs.

**FIG 8:**
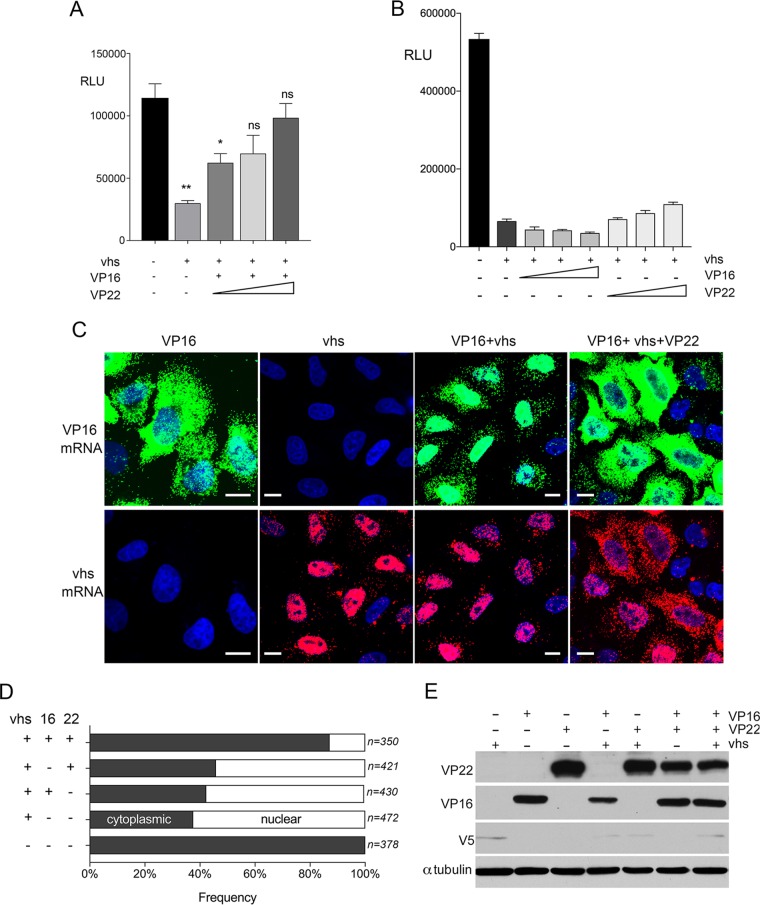
Coexpression of VP22 and VP16 with vhs rescues the cytoplasmic localization of mRNAs and PABP but not vhs translation. (A and B) HeLa cells were transfected with a plasmid expressing GLuc, together with the indicated plasmids. After 20 h the medium was changed and then harvested 3 h later, and the level of GLuc was measured. The mean ± standard error of the data from one representative experiment is given (*n* = 3). Statistical analysis was carried out using an unpaired, two-way Student's *t* test. *, *P* < 0.05; **, *P* < 0.01. (C) HeLa cells transfected with the denoted plasmids were processed for mRNA FISH with probes for VP16 and vhs transcripts. Bars = 10 μm. (D) HeLa cells transfected with the denoted plasmids were fixed at 20 h and processed for immunofluorescence with the PABP antibody. The cells were scored for the nuclear or cytoplasmic localization of PABP (*n* = number of cells counted). (E) HeLa cells transfected with the denoted plasmids were lysed 36 h later and analyzed by Western blotting with the indicated antibodies.

We next examined whether coexpressed VP22 and VP16 could influence the nuclear retention of mRNA by vhs. Using the GLuc reporter, we first tested the ability of cotransfected VP22- and VP16-expressing plasmids to rescue expression of the GLuc reporter in the presence of vhs. HeLa cells were transfected with the plasmids expressing GLuc, vhs, and VP16, together with increasing amounts of VP22-expressing plasmids (Fig. 8A). This revealed that GLuc expression was rescued when an increasing amount of VP22-expressing plasmid was added (Fig. 8A), while FISH studies on cells cotransfected with all three plasmids also indicated that VP22 restored VP16 and vhs transcripts to cytoplasmic puncta similar to those seen for translating mRNAs (Fig. 8C). This restoration of vhs and VP16 transcripts to the cytoplasm in the presence of the vhs, VP22, and VP16 proteins also correlated with a substantial increase in the percentage of cells where PABP was cytoplasmic (Fig. 8D). In line with the findings of a previous study (28) and the fact that vhs mRNA was now present in cytoplasmic puncta, we next tested the ability of VP16 and VP22 to together increase the translation of vhs by cotransfection of plasmids expressing V5-vhs, VP22, and VP16. However, in our hands this coexpression failed to enhance vhs protein levels in any number of assays that we carried out in HeLa cells (Fig. 8E), Vero cells, or COS cells (not shown), suggesting that even when localized in a cytoplasmic location similar to that of mRNAs that are being actively translated, the vhs mRNA is inherently untranslatable.

### Identification of a *cis*, transferable signal in the vhs mRNA that inhibits translation.

**FIG 9:**
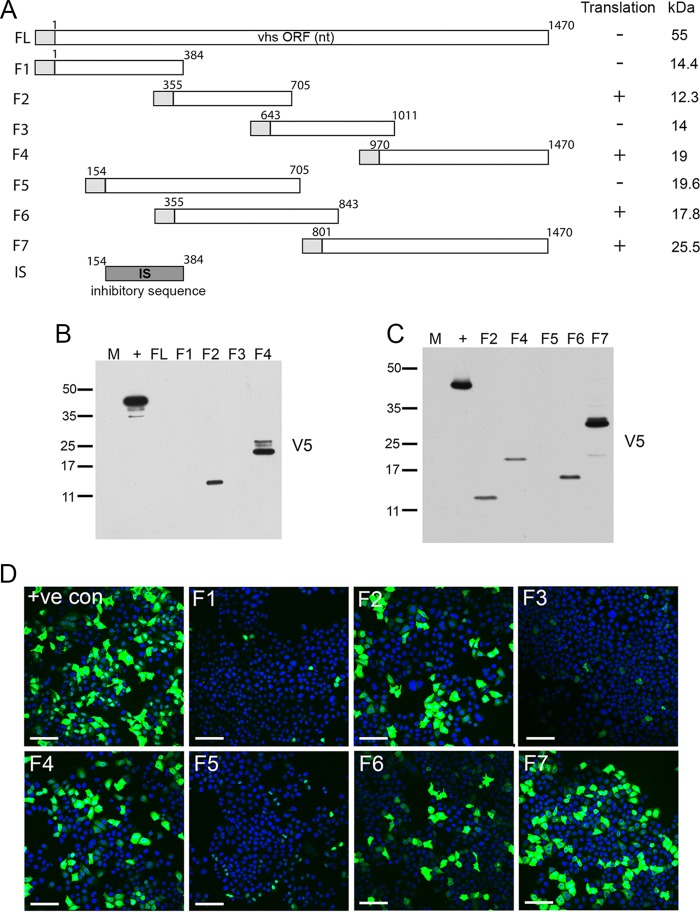
The vhs mRNA contains a region(s) that inhibits translation. (A) Line drawing of full-length (FL) vhs mRNA, showing the subdomains that were to be translated as V5 fusion proteins. Successful detection by Western blotting and the predicted sizes are summarized on the right-hand side. Numbers refer to nucleotides (nt). Gray boxes, V5 tag. (B and C) HeLa cells were transfected with plasmids expressing V5-tagged F1, F2, F3, F4, or full-length vhs (B) or V5-tagged F2, F4, F5, F6, F7, or full-length vhs (C), and total lysates were subjected to Western blotting with the V5 antibody. Lane M, mock-transfected cells; lane +, control cells with V5-tagged UL16. The numbers to the left of the gels in panels B and C are molecular masses (in kilodaltons). (D) HeLa cells transfected with plasmids expressing V5-tagged control or V5-tagged vhs fragments were processed for immunofluorescence with the V5 antibody (green) and counterstained with DAPI (blue). +ve con, positive control. Bars = 100 μm.

Given the inherent repression of vhs mRNA translation, even when localizing to cytoplasmic puncta, we reasoned that the vhs open reading frame itself may contain a region that inhibits its translation. Four overlapping regions covering the entire vhs mRNA (Fig. 9A) were next expressed as individual V5 fusion proteins in HeLa cells and tested by Western blotting. This indicated that regions F2 and F4 of vhs were efficiently translated, but regions F1 and F3 were not (Fig. 9B). To further refine the inhibitory sequences, we tested three further overlapping V5-tagged peptides (F5, F6, and F7 in Fig. 9A), which revealed that F5 was not translated, whereas both F6 and F7 were (Fig. 9C). The relative expression of each of these vhs fragments was also confirmed by immunofluorescence with the V5 antibody (Fig. 9D). Hence, an ∼230-nucleotide domain within the F1 region of the vhs open reading frame appears to be involved in inhibiting translation (Fig. 9A, inhibitory sequence [IS]).

**FIG 10:**
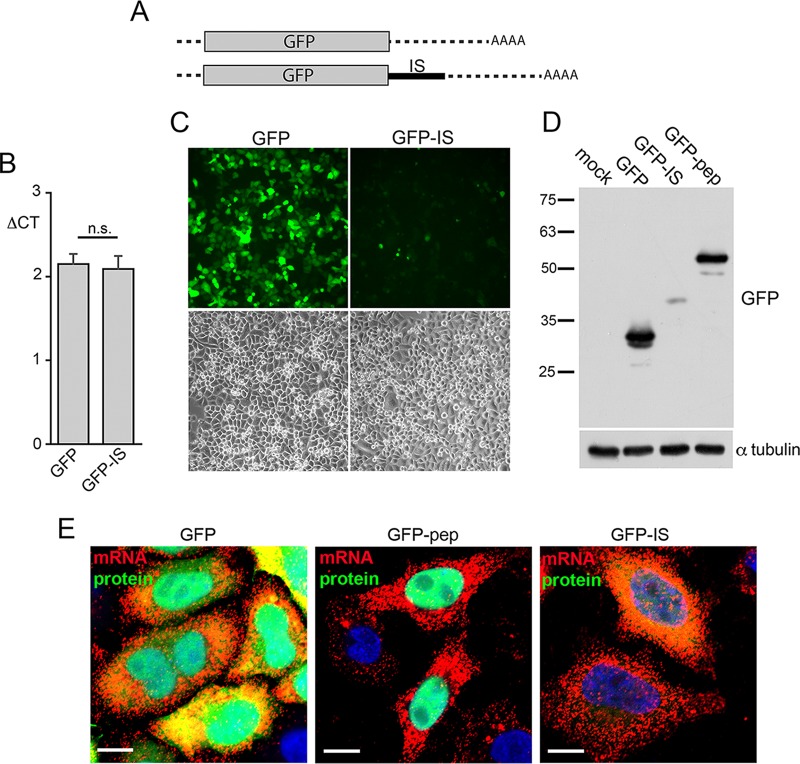
The vhs inhibitory sequence is transferable. (A) Line drawing showing the insertion of the predicted minimal inhibitory sequence (IS) in frame with the GFP open reading frame of pEGFPC1. (B) Total RNA was extracted from HeLa cells transfected with plasmids expressing either GFP or GFP-IS and subjected to qRT-PCR using primers specific for GFP. Results are shown as the GFP mRNA level relative to the level of the 18S RNA reference gene (Δ*C_T_*). The mean ± standard error of the data from one representative experiment is given (*n* = 3). Statistical analysis was carried out using an unpaired, two-way Student's *t* test. (C) HeLa cells transfected with plasmids expressing GFP or GFP-IS were imaged live using the same camera settings for each sample. (D) HeLa cells transfected with plasmids expressing GFP, GFP-IS, or a control GFP fusion protein (GFP fused to a 100-residue peptide from UL47) were analyzed by SDS-PAGE and Western blotting with an antibody against GFP. The numbers to the left of the gels are molecular masses (in kilodaltons). (E) HeLa cells were transfected with plasmids expressing GFP, GFP-IS, or a GFP control fusion peptide (GFP-pep). Following fixation and treatment with DNase, the cells were processed for RNA FISH with a GFP probe (red) and counterstained with DAPI (blue). In each case, GFP fluorescence was imaged using the same microscope setting. Bars = 10 μm.

This minimal inhibitory sequence (IS) region of vhs was inserted behind the open reading frame for GFP to determine if it could inhibit the translation of a chimeric transcript (Fig. 10A). HeLa cells transfected with plasmids expressing GFP or GFP-IS were first analyzed by qRT-PCR for GFP mRNA, indicating that there was no significant difference in transcription from the two plasmids (Fig. 10B). Nonetheless, live cell imaging indicated a profound reduction in GFP fluorescence in GFP-IS-transfected cells compared to GFP-transfected cells (Fig. 10C), as confirmed by Western blotting (Fig. 10D). This reduction was not simply a consequence of adding a peptide to GFP, as evidenced by expression from an alternative GFP fusion protein (Fig. 10D). FISH for GFP mRNA expressed from the same plasmids revealed that the IS—which, as shown above, inhibits GFP translation—had no discernible effect on GFP mRNA localization (Fig. 10E). Thus, the short discrete sequence at the 5′ end of the vhs mRNA can inhibit translation of a heterologous transcript without altering its localization. In short, even though vhs mRNA can be targeted to apparent translation sites in the cytoplasm by a VP16-VP22-mediated process, its translation is still inhibited via the presence of at least one inhibitory domain within the vhs-coding sequence.

## DISCUSSION

*In vivo* studies have shown that the vhs endoribonuclease of HSV-1 is important for pathogenesis of the virus, despite being nonessential for virus replication in culture (41, 42). In this respect, its role in degrading cellular transcripts that encode antiviral factors is likely to be vital for counteracting host responses to infection (20). However, given its destructive function, vhs, like other virus-encoded endoribonucleases, is a protein whose activity must be carefully regulated during virus infection, and it has been suggested that if left unchecked, vhs could be lethal to HSV-1 replication, causing degradation of virus transcripts and subsequent shutdown of virus protein translation. We show here that translation of vhs is regulated by a complex interplay of posttranscriptional mechanisms of both cellular and viral origin, which point to the lethality of unrestrained expression and the necessity to maintain a balance between translation of cellular and viral transcripts. This regulation is underpinned but not solely explained by aberrant localization of the vhs mRNA.

During infection, vhs mRNA was found in cytoplasmic puncta similar to those for other viral mRNAs. Nonetheless, when expressed alone, it tended to accumulate in cytoplasmic granules, where no translation was evident. Differential localization of mRNA is an efficient means of translational control in the cell and is used to ensure that mRNAs are translated not only in the correct location but also at the time that they are needed (43). Although these vhs-containing RNA granules are not stress granules (data not shown), it is likely that they represent some cellular mechanism for sequestering mRNA away from translating ribosomes and may form during transfection because, as our data indicate, vhs mRNA is inherently untranslatable in the absence of additional factors provided in *trans* by virus infection, due to the presence of at least one inhibitory sequence in the vhs open reading frame. A discrete 200-nucleotide motif from close to the 5′ end of the vhs transcript was transferable to a heterologous mRNA, inhibiting its translation while maintaining the punctate localization seen for actively translating mRNAs. The presence of a second IS was also inferred from the fact that the F3 region of vhs was similarly inhibited for translation (Fig. 9B and D). Similar inhibitory sequences have been found along the highly structured coding regions of a number of positive-strand RNA virus genomes and cellular transcripts, and these require cellular helicases to relieve inhibition (44). Hence, the secondary structure within the vhs open reading frame may make this transcript refractory to ribosome processivity. While it is formally possible that this inhibitory sequence could globally inhibit translation, this is considered unlikely, given the fact that our results suggest that shutoff of translation may be due to nuclear retention of mRNAs (see below). The lack of vhs translation in Δ22-infected cells and transfected cells confirmed that VP22 is required but not sufficient to overcome this translational block. Nonetheless, to date we have been unable to identify a second partner of VP22 that is required for vhs translation, as analysis of vhs expression in virus mutants in other known components of the VP22 interactome—VP16, gE, gM, ICP0, and UL47—failed to identify another factor that was required for vhs translation.

Because of the dual localization of vhs mRNA in transfected cells, we propose that although it is inherently untranslatable, an unknown stimulus (for example, cell cycle-related expression of factors required for vhs translation) relieves the inhibition of vhs translation in a subpopulation of cells, leading to degradation of both its own and other cytoplasmic mRNAs, thereby freeing PABP to relocalize to the nucleus, where it is subsequently retained, as described in other systems (38, 40, 45). As shown here, expression of vhs is required in HSV-1 infection and is sufficient, when expressed in isolation, to cause nuclear retention of PABP. Compelling studies from the Glaunsinger group have implicated hyperadenylation in a block to the nuclear export of mRNAs in the presence of the KSHV Sox protein (37, 38). Moreover, the same group has shown that Sox-induced degradation of cytoplasmic mRNAs leads to negative feedback on RNA polymerase II transcription in the nucleus (46). These results reveal a coupling between mRNA degradation in the cytoplasm and mRNA transcription and processing in the nucleus, and taken together with a previously reported reduction in RNA polymerase II activity on cellular genes in HSV-1-infected cells (47), they suggest that the vhs-specific reduction in cellular mRNAs may reflect a combination of activities over and above transcript stability. Notwithstanding this complex process, the ultimate effect of such an autoregulated negative feedback loop would be to efficiently limit the extent of vhs endoribonuclease activity present in the cytoplasm while successfully blocking translation of multiple other mRNAs by nuclear entrapment. It could therefore be argued that the main activity of vhs, at the very least, when expressed in isolation, is not in mRNA degradation, which is somewhat limited in this scenario, but, rather, is in blocking mRNA export from the nucleus, leading to a global reduction in translation.

Both VP22 and vhs interact directly with different regions of VP16, with the accepted model being that this trimeric complex inhibits vhs endonuclease activity later in infection, allowing translation of virus proteins (25, 26). We present evidence here that late virus transcripts, including vhs itself, are partially retained in the nuclei of HeLa cells infected with HSV-1 lacking VP22, suggesting a potential role for VP22 in overcoming vhs-induced nuclear retention of viral transcripts. Indeed, our studies from transfected cells revealed that coexpression of VP22 and VP16 relieved the vhs-induced nuclear retention of vhs mRNA, coexpressed mRNAs, and PABP, suggesting that the ultimate role of this trimeric complex could be to regulate the export of mRNA for translation late in infection. Although it has been shown in other studies that cells infected with Δ22 exhibit translational shutoff and tend to acquire a secondary mutation of vhs to allow viability (22, 27), our own Δ22 virus replicates efficiently in Vero cells and has a Wt vhs sequence (30) that, as presented here, is functional in reducing cellular mRNA levels during infection yet is no more active against the transcripts tested here than in Wt infection. Moreover, its effect on virus transcript levels is limited, and the levels of all virus proteins tested here—with the exception of vhs itself—were close to Wt levels, suggesting that vhs endoribonuclease activity is not overactive in Δ22-infected HeLa cells. The difference between this Δ22 virus phenotype and that of a virus lacking VP16, which exhibits a relatively early and complete shutoff of virus translation that is incompatible with virus replication in culture (23, 48), could be explained by the relative levels of vhs protein present in these infections. Hence, in the absence of VP22, expression of vhs is attenuated and its activity would be restricted accordingly, while in the absence of VP16, vhs is likely to be expressed at Wt levels, such that its activity could be lethally high and prohibit any translation during infection.

In summary, our studies presented here add a significant new understanding to the regulation of the expression of a viral endoribonuclease which has implications over and above virus gene expression in the infected cell. The cell and virus are shown to work in combination to carefully control the posttranscriptional fate of the vhs mRNA and ensure that the vhs protein is produced in a regulated and nonlethal fashion. Moreover, the hypothesis that vhs, VP16, and VP22 together regulate mRNA export from the nucleus late in infection opens up a new avenue of exploration for this enigmatic virus-encoded complex.

## MATERIALS AND METHODS

### Cells and viruses.

Vero and HeLa cells were cultured in Dulbecco modified Eagle medium (DMEM) supplemented with 10% newborn calf serum (NCS). Viruses were routinely propagated in Vero cells, with titrations carried out in DMEM supplemented with 2% human serum. HSV-1 strain 17 (s17) was used routinely. The s17-derived VP22 deletion mutant (Δ22) has been described before (49). Also, the vhs knockout virus (Δvhs) has been described before (15). HSV-1 strain Kos with a deletion of the C-terminal 36 residues of VP16 (3v) and its revertant (3vR) have been described elsewhere (50) and were kindly provided by Steve Triezenberg (Van Andel Institute). Kos expressing mCherry in place of UL47 was kindly provided by Colin Crump. The construction of viruses expressing GFP-tagged VP22 (GFP from residues 1 to 301 [GFP1-301]) and GFP-tagged VP22 subdomains (GFP192-301, GFP108-301, GFP1-212, and GFP1-165) has been described previously (51, 52). All viruses used in this study are presented in Table 1.

**TABLE 1:** Viruses used in this study

Virus strain	Derived from:	Genotype (reference)
s17		Wt
Δ22	s17	Expresses GFP in place of VP22 (49)
Δvhs	s17	LacZ inserted into the UL41 gene (15)
ΔICP0	s17	A 2-kb deletion in both copies of ICP0 (59)
GFP1-301	s17	GFP fused to full-length VP22 (51)
GFP1-212	s17	GFP fused to residues 1–212 of VP22 (52)
GFP1-165	s17	GFP fused to residues 1–165 of VP22 (52)
GFP108-301	s17	GFP fused to residues 108–301 of VP22 (52)
GFP160-301	s17	GFP fused to residues 160–301 of VP22 (52)
GFP192-301	s17	GFP fused to residues 192–301 of VP22 (52)
sc16		Wt
ΔgE	sc16	LacZ inserted into Us8 (60)
ΔgM	sc16	LacZ replaces codons 134–466 of UL10 (61)
ΔgEgM	sc16	LacZ inserted into UL10, luc expression cassette inserted into Us8 (62)
Kos		Wt
3v	Kos	Missing C-terminal 36 residues of VP16 (50)
3vR	Kos	Revertant of RP3v (50)
Δ47	Kos	mCherry in place of UL47 (a kind gift from Colin Crump)

### Antibodies.

VP22 (AGV031) and UL47 (5283) antibodies have been described elsewhere (53, 54). Other antibodies used in this study were kindly provided by the following individuals: gD (LP14), VP16 (LP1), and gB (R69), Tony Minson (University of Cambridge); ICP27, Steve Rice (University of Utah); vhs, Duncan Wilson (Albert Einstein College of Medicine); gE (3114), David Johnson (Oregon Health and Science University, Portland, OR, USA); and UL16 and UL21, John Wills (Pennsylvania State University). The V5, GFP, and α-tubulin antibodies were purchased from Abcam, Clontech, and Sigma, respectively. The antibodies to conjugated ubiquitin (FK2) and PABP were from Enzo Life Sciences and Santa Cruz, respectively. Horseradish peroxidase-conjugated secondary antibodies were from Bio-Rad Laboratories.

### Plasmids.

Plasmid expressing VP22 has been described before (55). VP16 was expressed in a transfection from pcVP16 in which the VP16 open reading frame had been inserted into pcDNA1 (Invitrogen), and GFP was expressed from plasmid pEGFPC1 (Clontech). A plasmid expressing glycoprotein D in pcDNA1 (pcDNAgD) was provided by Helena Browne (University of Cambridge). Plasmids expressing V5-tagged vhs from strain 17 or strain Kos or V5-tagged subdomains of vhs were constructed by PCR amplification of the denoted regions of the vhs gene using the primers shown in Table 2, followed by digestion with BglII and EcoRI and insertion into BamHI- and EcoRI-digested plasmid pCMV-V5 (56). Plasmid expressing V5-tagged UL16 was constructed in the same way following PCR amplification of the UL16 open reading frame. The vhs R257C variant (vhs*) was derived by PCR amplification of the UL41 gene from our Δ22 revertant virus genomic DNA (30), followed by insertion into the pCMV-V5 expression plasmid. A plasmid expressing GFP fused to the inhibitory sequence of the vhs gene was similarly constructed by PCR amplification and insertion into BglII- and EcoRI-digested pEGFPC1. Plasmid pcvhs, expressing untagged vhs, was similarly constructed by PCR amplification of the full-length vhs gene using the primers shown in Table 2, followed by insertion into HindIII- and XbaI-digested pcDNA1. Plasmids were transfected into HeLa cells using the Lipofectamine 2000 reagent (Invitrogen), and cells were routinely harvested 20 h later.

**TABLE 2:** Primers for PCR amplification of vhs and regions therein

Construct	Sequence^*a*^	
	Forward	Reverse
pcvhs	**GCAAGCTT**ACCATGGGTTTGTTCGGGATGA	**GCTCTAGACTA**CTCGTCCCAGAATTTGG
V5-vhs	**GCAGATCT**ATGGGTTTGTTCGGGATGATG	**GCGAATTCCTA**CTCGTCCCAGAATTTGG
V5-F1	**GCAGATCT**ATGGGTTTGTTCGGGATGATG	**GCGAATTCCTA**TCTGGAGTCGGTGATGGGG
V5-F2	**GCAGATCT**AGACCGCCTTCCCCCATCA	**GCGAATTCCTA**GATCGTGGGAATGTAGCAGG
V5-F3	**GCAGATCT**GACCTCCTGTTGATGGGCT	**GCGAATTCCTA**CTCGTCGTCTTCGTATCCG
V5-F4	**GCAGATCT**ATTCTAACCCAACAGATCGCC	**GCGAATTCCTA**CTCGTCCCAGAATTTGG
V5-F5	**GCAGATCT**AGTTACGACCGCGAGGCCA	**GCGAATTCCTA**GATCGTGGGAATGTAGCAGG
V5-F6	**GCAGATCT**AGACCGCCTTCCCCCATCA	**GCGAATTCTCA**GGTCCAGTGACATTCGC
V5-F7	**GCAGATCT**TACGCCTCCGTGGAGGATG	**GCGAATTCCTA**CTCGTCCCAGAATTTGG
GFP-IS	**GCAGATCT**AGTTACGACCGCGAGGCCA	**GCGAATTCCTA**TCTGGAGTCGGTGATGGGG

a Boldface represents restriction sites used for cloning of the DNA fragments.

### SDS-PAGE and Western blotting.

Protein samples were analyzed by SDS-polyacrylamide gel electrophoresis (PAGE) and were either stained with Coomassie blue or transferred to a nitrocellulose membrane for Western blot analysis. Western blots were developed using SuperSignal West Pico chemiluminescent substrate.

### GFP-TRAP pulldown assay.

A GFP trap kit (Chromotek) was used to purify GFP-tagged protein from infected cells according to the instructions in the manufacturer's manual. Briefly, 5 × 10^6^ confluent cells were infected with virus expressing a GFP at a multiplicity of infection of 1. After 16 h, the infected cells were harvested and lysed for 20 min on ice in 1 ml radioimmunoprecipitation assay buffer (50 mM Tris, pH 7.5, 150 mM NaCl, 0.1% SDS, 1% Na deoxycholate, 1% NP-40, protease inhibitors [Roche]). The cell lysates were centrifuged, and the supernatants were incubated with 15 μl washed GFP-TRAP beads for 30 min at 4°C with rotation. The beads were washed twice and resuspended in loading buffer for analysis by SDS-PAGE.

### Immunoprecipitation assay.

Cells grown in 6-cm dishes were infected with the relevant viruses at a multiplicity of infection of 1, and immunoprecipitation was carried out as described previously (57).

### qRT-PCR.

Total RNA was extracted from cells using a Qiagen RNeasy kit. Excess DNA was removed by incubation with DNase I (Invitrogen) for 15 min at room temperature, followed by inactivation for 10 min at 65°C in 25 nM EDTA. A SuperScript III system (Invitrogen) was used to synthesize cDNA using random primers according to the manufacturer's instructions. All quantitative RT-PCR (qRT-PCR) assays were carried out in 96-well plates using a Mesa Blue quantitative PCR MasterMix Plus for SYBR assay (Eurogentec). The primers for cellular and viral genes are shown in Table 3. Cycling was carried out in a LightCycler instrument (Roche), and relative expression was determined using the ΔΔ*C_T_* threshold cycle (*C_T_*) method (58), using 18S RNA as a reference. The mean for replicate changes in *C_T_* (Δ*C_T_*) values was used to generate a single ΔΔ*C_T_* value. The standard error of the mean (SEM) was then calculated using the equation [SE(ctrl)^2^ + SE(trt)^2^], where SE(ctrl) is the standard error for control cells and SE(trt) is the standard error for treated cells. Statistical analysis was carried out using an unpaired, two-way Student's *t* test.

**TABLE 3:** Primers used for quantitative RT-PCR

Gene	Sequence	
	Forward	Reverse
VP22	CGCGATGAGTACGAGGATCT	GAGGGCATAATCCGACTCGT
ICP27	GATCGACTACGCGACCCTTG	GCAGACACGACTCGAACACT
vhs	ATCCAACACAATATCACAGCCCATCAACAG	CGCCAACCTCTATCACACCAACACG
VP16	TAACCGTCTCCTCGACGACT	CTGGGCAGCGTTGATAGGAA
UL47	GCATCCGCCAAAAAGCTCAT	GGTATATCACGGGCGATGGG
UL16	GTCGTAACGGAGTCCTGTCC	AGAGAAGCGGACACAGGCT
UL21	GAAGACACACGCTGTCCAGA	CCAAGTTTGGTCTGGTCGTT
gB	GTCTGCACCATGACCAAGTG	GGTGAAGGTGGTGGATATG
RPLP0	ACTCTGCATTCTCGCTTCCT	GGACTCGTTTGTACCCGTTG
GAPDH	ACAGTCAGCCGCATCTTC	CTCCGACCTTCACCTTCC
β-Actin	CTGGACCGGTCAAGGTGACA	AAGGGACTTCCTGTAACAATGCA
GFP	GAAGCAGCACGACTTCTTCAA	AGTCGATGCCCTTCAGCTC
18S RNA	CCAGTAAGTGCGGGTCATAAGC	GCCTCACTAAACCATCCAATCGG

### Immunofluorescence.

Cells grown on coverslips were treated as described previously (57). Images were acquired on a Nikon A2 confocal microscope and processed using Adobe Photoshop software.

### FISH of mRNA.

Cells were grown in 2-well slide chambers (Fisher Scientific) and either transfected with the relevant plasmid or infected with virus. At the appropriate time, cells were fixed for 20 min in 4% paraformaldehyde and then dehydrated by sequential 5-min incubations in 50%, 70%, and 100% ethanol. Fluorescent *in situ* hybridization (FISH) was then carried out using Applied Cell Diagnostics (ACD) RNAscope reagents according to the manufacturer's instructions. Briefly, cells were rehydrated by sequential 2-min incubations in 70% and 50% ethanol and phosphate-buffered saline (PBS) and treated for 30 min at 37°C with DNase, followed by treatment with protease for 15 min at room temperature. Cells were then incubated for 2 h at 40°C with the relevant RNAscope probe (VP16, ICP27, TK, gD, vhs, or GFP, as designed by ACD), followed by washes and amplification stages according to the manufacturer's instructions. After incubation with the final fluorescent probe, the cells were mounted in Mowiol mounting medium containing DAPI (4′,6-diamidino-2-phenylindole) to stain the nuclei, and images were acquired with a Nikon A2 inverted confocal microscope.

### Gaussia luciferase (GLuc) reporter assay.

HeLa cells were transfected with plasmid pCMV-GLuc-1, and 24 h later, the medium was replaced. After 3 h, the extracellular medium was collected and chemiluminescence was measured by injection of coelenterazine at 1 μg/ml in PBS and read on a Clariostar plate reader.
